# Pharmacogenetic testing and counselling in the community pharmacy: mixed-methods study of a new pharmacist-led service

**DOI:** 10.1007/s11096-023-01596-8

**Published:** 2023-06-20

**Authors:** Chiara Jeiziner, Henriette E. Meyer zu Schwabedissen, Kurt E. Hersberger, Samuel S. Allemann

**Affiliations:** 1 Pharmaceutical Care Research Group, Department of Pharmaceutical Sciences, University of Basel, Klingelbergstrasse 50, 4056 Basel, Switzerland; 2 Biopharmacy, Department of Pharmaceutical Sciences, University of Basel, Klingelbergstrasse 50, 4056 Basel, Switzerland

**Keywords:** Interprofessional pharmaceutical care, Medication review, Personalized pharmacotherapy, Pharmacy service

## Abstract

**Background:**

Pharmacogenetic (PGx) testing and counselling (short: PGx service) in the community pharmacy is not routinely practiced. We propose a comprehensive pharmacist-led service where PGx information is integrated into medication reviews.

**Aim:**

To evaluate the pharmacist-led service comprising PGx testing and counselling (PGx service) from the perspective of patients.

**Method:**

For this mixed-methods study, we conducted two follow-up interviews *F*1 and *F*2 with patients recruited for the PGx service in a community pharmacy after 1st of January 2020. The semi-structured interviews were held by phone call and covered understanding of PGx, the implementation of recommendations, handling of PGx documents (list of concerned substances and PGx recommendation), gain in medication knowledge, and willingness to pay for the PGx service.

**Results:**

We interviewed 25 patients in *F*1 and 42 patients in *F*2. Patients were generally able to understand and use results of the PGx service. At least one PGx recommendation was implemented for 69% of the patients. Handling of PGx documents ranged from patients having forgotten about the PGx results to patients consulting the list for every medication-related decision; the latter often expecting negative effects. Finally, 62% of the patients were willing to pay for the PGx service.

**Conclusion:**

For future PGx testing and counselling, HCPs should consider the patients’ health literacy in a standardized way and use adequate communication skills to enhance the patient's understanding in PGx and to attenuate potential negative expectations.

## Impact statements

- Evaluation of a new pharmacist-led service comprising pharmacogenetic (PGx) panel testing covering up to 30 genes and 100 variations integrated into a comprehensive medication review.
- Our study covers the broad patient population which is encountered in the community pharmacy.
- We used a mixed-methods study to evaluate the new pharmacist-led PGx service from the perspective of patients.
- In more than two-thirds of the patients, at least one PGx recommendation was implemented.
- When communicating PGx results, healthcare professionals need adequate communication skills to attenuate potential negative expectations towards the medication.

## Introduction

In clinical practice, interindividual drug response ranges from ineffectiveness to adverse drug reactions (ADRs). Pharmacogenetics (PGx) is the study of genetic variations related to drug response [1], i.e., activity and/or expression of enzymes and transporters involved in drug metabolism. Of 167 substances containing information on PGx influencing drug safety and/or efficacy in Swiss drug labels, 55% (93) are classified as “actionable” PGx information [2], thereby referring to changes in efficacy, dosage, metabolism or toxicity due to genetic variations without mentioning the requirement for a genetic test [3]. Furthermore, international recommendations on PGx-guided drug selection and dosing are available today [4, 5]. However, PGx is not yet routinely used in neither primary nor secondary care. There are numerous barriers to the adoption of PGx ranging from lack of education to the reluctance of health insurances to reimburse healthcare professionals (HCPs) for unacknowledged procedures [6–8]. Nevertheless, there are also clear enablers, such as accumulating evidence about clinical utility of PGx and the option of putting the pharmacist in the role of providing a PGx service [6, 9]. Notably, the application of a PGx panel test offers the possibility to counsel on several drugs and not only one.

**Fig. 1:**
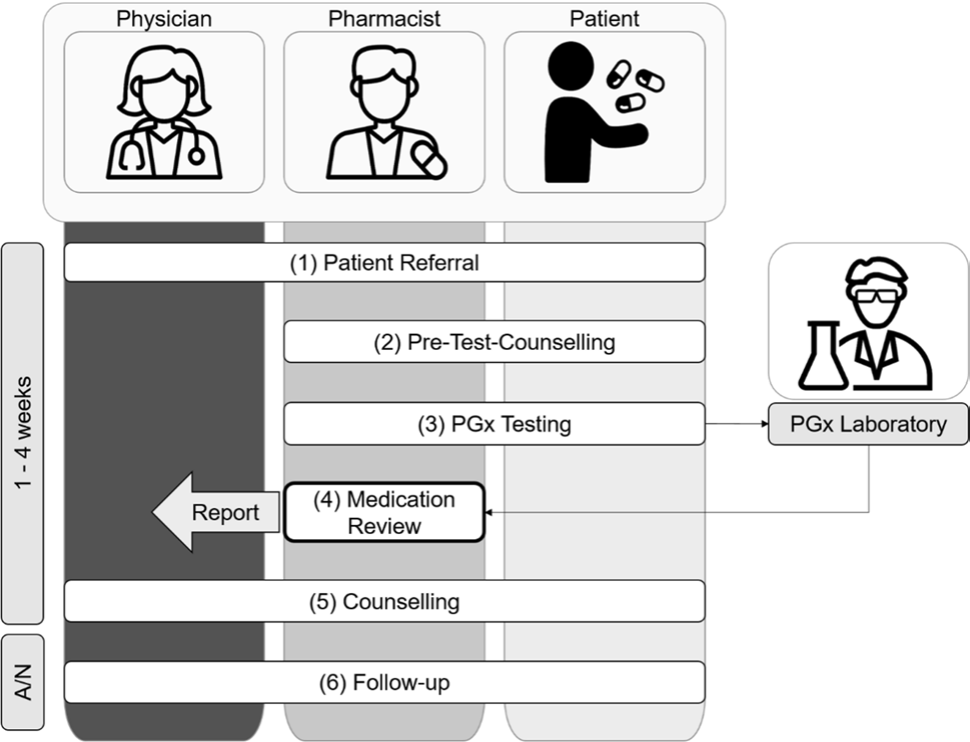
Overview of pharmacist-led service PGx testing and counselling (short: PGx service) [10]

We performed a case series (Clinicaltrials.gov: NCT04154553) where more than 100 patients experiencing ADRs and/or therapy failure (TF) with substances known to be affected by PGx were recruited for pharmacist-led PGx testing and counselling (short: PGx service) [10, 11]. The comprehensive pharmacist-led PGx service is depicted in Fig. 1. For the PGx service, we worked with a commercial provider offering PGx panel testing covering up to 30 genes and 100 variations together with evidence-based interpretation. The resulting recommendations for a single drug in view of the individual genotype are categorized as “Hinweis” (*Engl*.: indication, problems could arise and careful monitoring is needed), “Verdacht” (*Engl*.: suspicion, high probability for problems, change of dose or drug needed) or “Gefahr” (*Engl*.: danger, risk for an acute problem, drug to be avoided or used with ultimate precaution and/or dose adaption). To ease understanding by the patient, a traffic light system was used to visualize medications with “indication” in yellow, “suspicion” in orange, and “danger” in red. Moreover, the service comprises a complete profile of the 30 tested genes and their variants as well as an individualized list of concerned substances. The patient received the list of concerned substances (list of all substances, which are included in the data bank coded with a traffic light system [yellow, orange, red] according to the individual’s pharmacogenetic profile), and an individualized PGx recommendation (written report from the pharmacist).

Patients’ perspectives on commercial PGx panel testing have been evaluated with the call for further research [12–14]. If a pharmacist provides a pharmacist-led service involving PGx testing and counselling, the patient needs to be able to understand, and in the following, implement the PGx-based recommendations.

### Aim

As part of the case series study, we aimed to evaluate the patients’ perspective of the pharmacist-led service comprising PGx testing and counselling. The service included a follow-up. In this study, we conducted semi-structured interviews with counselled patients one month and at least four months after the PGx service. Patients’ understanding of PGx, implementation of recommendations, handling of PGx documents (list of concerned substances and PGx recommendation), gain in medication knowledge, and willingness to pay for the PGx service were collected.

### Ethics approval

The case series “Pharmacogenetic Testing of Patients with unwanted Adverse Drug Reactions or Therapy Failure” was performed in line with the principles of the Declaration of Helsinki. Approval was granted by the local ethics committee in northwestern and central Switzerland (Ethikkommission Nordwest- und Zentralschweiz, Hebelstrasse 53, 4056 Basel, eknz@bs.ch) (EKNZ-2019–01,452) on 31.10.2019.

## Method

For the elaboration and reporting of our study, we considered the COREQ (Consolidated criteria for Reporting Qualitative research) checklist [15].

### Study design and setting

We used a mixed-methods study design [16, 17] with predefined themes aiming to explore patients’ understanding of PGx, implementation of recommendations, handling of PGx documents (list of concerned substances and PGx recommendation), gain in medication knowledge, and willingness to pay for the PGx service. The PGx service took place in a community pharmacy in Basel. We conducted two semi-structured interviews with patients one month (*F*1) and 4 months or more (*F*2) after the PGx service. The use of both quantitative and qualitative data enabled a broad insight into the perspective of the patient.

### Participant recruitment

All patients recruited into the case series after December 1st 2020 were included for *F*1 and *F*2. In addition, and to increase the sample size, all patients recruited to the case series from January 1st 2020 to November 30th 2020 were included for *F*2. To check for data saturation, an interim analysis was conducted on September 30th 2021.

### Data collection

For the semi-structured interviews, CJ, PhD, female, a pharmacist by training who had met all participants in the case series previously, developed a separate interview guide for each of the two follow-up interviews, which were reviewed by two members of the PGx expert team (KH and HMzS) and piloted with five patients each. Data were collected by one interviewer (CJ) during bilateral phone calls and documented in a MS word template. During follow-up interview 1 (*F*1), patients answered six closed questions and three assessments via a 10-item Likert-scale in three sections about understanding of PGx, medication knowledge, and general feedback on the PGx service. During follow up-interview 2 (*F*2), patients answered 11 closed questions, three assessments via a 10-item Likert scale, and five open questions in four sections about PGx-related medication changes, handling of PGx-documents, medication knowledge, and willingness to pay for the PGx testing and counselling after having experienced the pharmacist-led service free of cost. For the latter, we explained to the patients the split in costs for the laboratory test (400 EUR) and costs for the counselling (300 EUR) including a first and second visit of 30 min each, sample collection, and preparation of the recommendation letter of at least 40 min. The 10-item Likert-scales were defined as follows:

- Appropriateness/comprehensibility:
  0 = “not at all appropriate/comprehensible” to 10 = “fully appropriate/comprehensible”
- Clarity: 0 = “not at all clear” to 10 = “fully clear”
- Usability: 0 = “not at all usable”10 = “fully usable”

To categorize the medication, we used the first level of the Anatomical Therapeutic Chemical (ATC) classification system.

Interview guides are available on request from the authors.

### Data analysis

The qualitative and quantitative elements of the mixed-methods study are presented in a convergent form for each of the interviewed themes. We analysed the data as follows:

- For quantitative data, we calculated proportions for two-point questions and medians for the 10-item Likert scales. For one open question in *F*1 and *F*2, the interviewer had to note if the patient had mentioned the list of concerned substances and/or the PGx recommendation or not.
- For qualitative data, we used the process of quantitizing, i.e. we transformed the quotes into numeric variables for comparison with the quantitative data [18]. Therefore, CJ and KH defined different categories based on answers to questions from follow-up interviews 1 and 2. Subsequently, three PGx experts (CJ, HMzS, AS) independently categorized the text answers according to the defined categories. In case of discrepancies between the three PGx experts, a discussion was held until consensus was found. Illustrative patient quotations were reported with the corresponding patient identifier, birth year, and sex. A description of the categorization is available on request from the authors.
- Finally, we reported qualitative data in narrative form (e.g., quotations) to enrich quantitative data.

## Results

We interviewed 25 of 26 approached patients for the first follow-up interview (*F*1) and 42 of 47 approached patients for the second follow-up (*F*2). The characteristics of patients interviewed in *F*1 and *F*2 were comparable in gender and age (Table 1). The broad range of 120 to 429 days since the second visit for *F*2 was due to the inclusion of patients that had been recruited into the case series study before Dec 1st 2020.

**Table 1:** Patient characteristics and specifications of follow-up interviews 1 (*F*1) and 2 (*F*2)

Characteristics	Follow-up interview 1 (*n *= 25)	Follow-up interview 2 (*n* = 42)
Female gender	17 (68%)	31 (74%)
Median age (IQR) [years]	56 (41–71); range: 18–79	54 (45–69.75); range 27–89
Mean time since 2nd visit [days]	47; range: 21–85	22;5 range: 120–429
Time period of recruitment to the case series study	Dec 1st 2020 to Sept 30th 2021	Jan 1st 2020 to Sept 30th 2021
Mean interview duration [min]	9; range: 6—22	22; range: 12–36 min

### Understanding of PGx (F1)

We interviewed two (6%) patients with only compulsory education, 11 (44%) patients with secondary level of education, and 12 (48%) patients with tertiary level of education. Patients valuated the language used by the pharmacist in the first and second visit as appropriate and the explanations of the PGx results as comprehensible on a 10-item Likert scale. In consequence, most patients (80%) felt no need for a further consultation.

By quantitizing the question “How would you explain the result of the pharmacogenetic test to a friend?”, we deduced the understanding of patients. Depending on the explanations provided by the patients, we differentiated four groups as follows:

- very good understanding, e.g.

*“Through genetic information, I can see to which substances I react best, with which substances side effects could occur, and which substances are better suited. […] So I can start [a medication] with pre-knowledge or try again with a specific medication.” a210825, female, 2003*

- good understanding, e.g.

*“I would explain what it is about and if the person understands it, I would explain my case, namely that my drugs have been classified differently, that there is none that is dangerous, but with two drugs I have to be careful, and there is one drug that I should not take because I have certain genetic predispositions so that I metabolize it quickly.” a210907, male, 2001*

- partial understanding, e.g.

*“I cannot explain it scientifically. Due to genetic studies, they studied my medications, whereof two were not optimal.” a210223, female, 1947*

- no understanding, e.g.

*“It is difficult to make an own interpretation.” a210409, male, 1955*

**Fig. 2:**
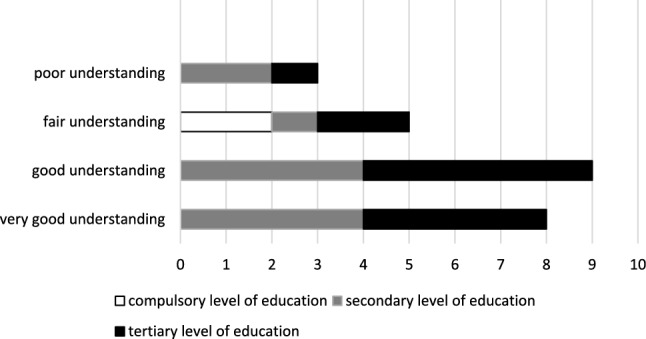
Level of understanding by the patient of the PGx results versus their level of education by number of individuals (*n* = 25)

In comparison with the reported level of education, patients with higher level of education tended to demonstrate a better understanding for PGx information (Fig. 2).

### Implementation of recommendations (F2)

*Referring physician* Half of the patients (*n *= 21) were referred by a general practitioner, whereas the other half were referred by a medical specialist. Thereof, rheumatologists (*n* = 11) and psychiatrists (*n* = 8) were the most frequent referring specialists. At the time of *F2*, 36 (86%) of the patients had a consultation with their referring physician since the second visit to the community pharmacy. Patients’ statements about the reaction of the referring physician differed from positive (*n* = 16, 38%), neutral (*n* = 7, 17%), negative (*n* = 4, 10%), or no statement (*n* = 15, 36%) because the physician did not take the time to consult the PGx documents or because consultation with the physician had not yet taken place.

*Other physician* 29 (69%) patients also visited other (non-referring) physicians and informed them of the PGx testing and provided them with the PGx recommendation. The three most frequent types of other physicians who had received PGx recommendations were general practitioners (*n* = 11), psychiatrists (*n* = 9), cardiologists (*n* = 4) as well as rheumatologists (*n* = 4). On average, recommendations were handed over to one (range: zero to five) other physicians.

Comparison of medication plans between current and the first visit revealed that, we identified a total of 75 changes with at least one implemented PGx recommendation for 29 (69%) of the patients. Of the 75 implemented PGx recommendations, almost half of them (*n *= 34) concerned substances of the anatomic group N “nervous system”. Part of these changes 16 (21%) comprised the start of new medication followed by a stop shortly afterwards.

### Handling of documents (F2)

The PGx recommendations were read by 35 (83%) of the patients and the clarity was rated with a median of 8 on a 10-item Likert. The list of concerned substances was used by 38 (90%) of the patients and the usability was rated with a median of 9 on a 10-item Likert scale.

**Fig. 3:**
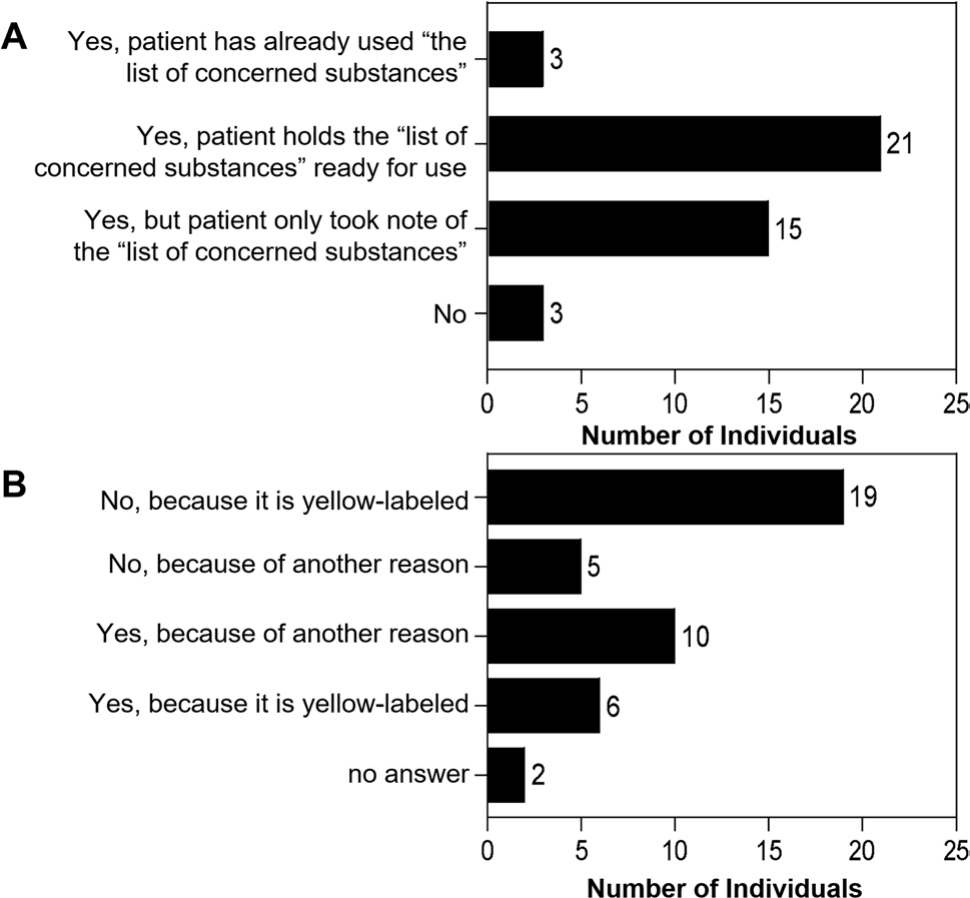
**A**: Use of the list of concerned substances; **B**: Purchase of ibuprofen in case of a yellow indication on the list of concerned substances (*n* = 42)

We asked patients if they had used the list of concerned substances (Fig. 3A). Depending on the context, in which the list of concerned substances was used, we differentiated four groups as follows:

- patients who have already actively made use of the list, e.g.

*“Whenever I get a new medication, I look at the list first. (Either the doctor already looks, or I point it out to him.) I keep the list in my purse.” a200205, female, 1962*

- patients who hold the list ready for use, e.g.

*“I looked at it and studied it, […] I also have it on my smartphone and consult it when a change is due.” a200618, female, 1970*

- patients who only took note of the list, e.g.

*“In the beginning…, I do not know in which drugs all these active ingredients are in. I read the list and saw what kind of drugs there are. I also looked at it together with the doctor. It should be him to give the necessary indication.” a201014, male, 1932.*

- patients who had not looked at the list of concerned substances. 
  Furthermore, patients were asked whether they would buy the over-the-counter drug ibuprofen if it were labelled with a yellow indication on their list of concerned substances, (Fig. 3B). Depending on the reason given for or against the purchase of ibuprofen, we differentiated four groups as follows:

- patients who would not buy it, because it is yellow-labelled, e.g.

*“After all, I have this clue that something else would probably work, which is why if there's something else, I'd rather buy something else.” a210601, male, 1989*

- patients who would not buy it, because of another reason, e.g.

*“Since I took Lamotrigine and Fycompa [i.e., perampanel], I am very careful, because both times I had an allergic reaction and once, I landed at the emergency ward.” a210525, female, 1980*

- patients who would buy it, because of another reason, e.g.

*“If I have made good experience so far [….] or no alternative is available. Otherwise, I would notice the side effects.” a200408, female, 1982:*

- patients who would buy it, because it is yellow-labelled, e.g.

*“Yellow is an indication, so I can take it if I need it. It is not orange, so it is no suspicion. I would be a little uncertain, but I would try it out.” a200124, female, 1971*

Patients that did not cite the list of concerned substances as a reason for or against the purchase of ibuprofen, provided numerous other reasons. Reasons for a purchase of ibuprofen comprised urgency in case of strong pain, willingness to try the substance and to give it a chance, good experiences before, the hope for an effect, or the awareness that its use would only be for a short term. Reasons against a purchase of ibuprofen comprised general scepticism towards medication, the wish to consult the physician prior to the intake of a new drug, the fear of interactions with drugs of the current regimen, or bad experiences with this particular drug (e.g., allergies).

### Gain in medication knowledge (F2)

**Fig. 4:**
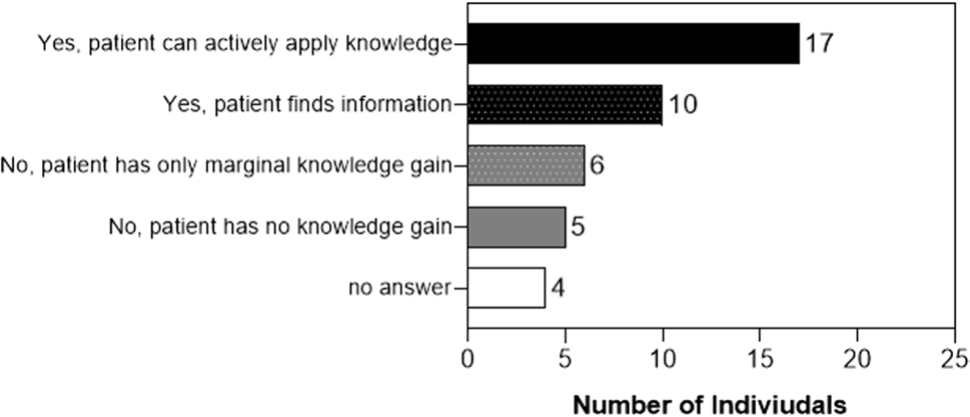
Gain in medication knowledge at follow-up interview 2 (*n* = 42)

If a new medication is suggested by the physician, 24 (57%) of the patients would consult their PGx documents (list of concerned substances/ PGx recommendation). For a further evaluation of the gain in medication knowledge, we asked patients whether they think they know better about their medications since the PGx service in *F2* (Fig. 4). We differentiated four groups as follows:

- patients who were able to actively apply their gained knowledge, e.g.

*“Yes, I now know differently about medication. The discussion with the pharmacist was useful. I have a better knowledge that certain medication groups work worse with me and that they are not so good for my body. That already helps me.” a201005, female, 1961.*

- patients who find the information, e.g.

*“It's interesting, it is clear to me. I do not understand so much, which is a pity, but I will always look from now on.” a210223, female, 1947*

- patients who only have marginal gain in knowledge, e.g.

*“Not better, but I no longer feel that I am crazy because I know that many [medication] is not so suitable for me…” 201,112, female, 1976*

- patients who had no gain in knowledge, e.g.

*“No, I do not think so.” a201110, female, 1979*

#### Willingness to pay (F2)

For most of the patients, both the laboratory (72%) and the counselling (76%) costs were adequate. A majority of the patients (62%) said that they would pay the complete cost for the PGx service. Lack of financial capacity was the most frequent reason for an inability to pay.

## Discussion

In this study, we evaluated a pharmacist-led service comprising PGx testing and counselling by conducting semi-structured interviews with counselled patients.

Similarly to our study, Martin et al. [14] assessed the perspective of 16 patients towards a pharmacist-provided PGx service in semi-structured interviews and identified four major themes, namely heterogeneity of patient PGx preferences and experiences, pharmacists as appropriate providers of a PGx service, considerations regarding the use of PGx results in routine healthcare, and perceived applications of PGx testing. These findings were confirmed in our study comprising 77 follow-up interviews.

In general, patients were able to understand PGx results. However, as most patients are unfamiliar with PGx testing, the language used during the communication of PGx results needs to be chosen very carefully [19]. When asking patients how they would explain the results of the PGx test to a friend, we recognized different levels of understanding. Accordingly, we could see a tendency of patients with high educational backgrounds to have a better understanding of PGx results. This observation corresponds with the literature showing that health literacy is positively associated with education [20]. Therefore, patients with higher education have a higher likelihood of accessing, understanding, appraising, and applying PGx information [21].

In our study, we saw that more than two thirds of the patients had at least one recommendation implemented. Almost half of the recommendations concerned substances of the nervous system, thereby confirming the need of PGx in the psychiatric setting [22, 23]. It has been shown that psychiatric patients are likely to benefit from a PGx test prior to the therapy to avoid ADRs or treatment failure [24, 25]. However, a part of the newly started medications was stopped shortly afterwards. Considering that 50% of patients with unipolar depression do not experience remission under a first-line antidepressant treatment [26], we claim that the addition of PGx information to therapeutic decision-making is substantial to help reduce the amount of trial and error regimens, even though PGx will not completely eliminate the percentage of unsuccessful regimens as there are other factors such as drug-drug interactions, medication adherence, and others influencing pharmacokinetics and pharmacodynamics of drugs.

The PGx recommendation as well as the list of concerned substances were evaluated as clear and usable for most patients. When asking patients about the list of concerned substances, more than half of them said that they held the list ready, and three patients reckoned they had already used it. As ibuprofen is the most frequently used Swiss drug known to be affected by PGx [27], we wanted to know how patients would proceed when they were confronted with a yellow indication for such a common drug on their list of concerned substances. A majority of the patients would use the list of concerned substances to decide for or against a purchase of ibuprofen. Only a small percentage would buy ibuprofen if it were yellow-labelled whereas a majority of the patients would not buy ibuprofen in the same situation. The latter patients seemed to be afraid of taking ibuprofen because of the indicated potential risk of ADRs or inefficacy. In 2009, Haga et al. [28] already postulated that PGx information could cause adverse effects or lack of adherence due to negative expectations triggered by the assumption that a drug could not work. Especially anxious patients might screen the list too critically and decide to a change their pharmacotherapy, thereby leading to wrong behaviour. On the one hand, a careful dose titration with the possibility of therapeutic drug monitoring is essential and on the other hand, HCPs need adequate communication skills to avoid potential negative expectations about the medication [29].

In our study, more than half of the patients reported that they would consult their PGx documents in case a new medication is suggested by the physician. Besides, more than half of the patients were able to actively apply their knowledge on PGx or were able to find the information on PGx. Both results show that patients experienced a gain in medication knowledge through the PGx service. It has been shown that patients perceive PGx testing as useful; however, the communication of PGx results still needs effort on the part of HCPs so that patients’ knowledge about their medication is increased [30].

In a systematic review by Hansen et al. [13], one of the main barriers mentioned is the cost of the PGx service. In our study, more than half of the patients would be willing to pay the estimated cost for the pharmacist-led PGx service comprising the laboratory costs and the counselling costs for a first and second visit of 30 min each, sample collection, and preparation of the recommendation letter of at least 40 min. Similar willingness to pay for a medication review with an average duration of 20 min was observed in an earlier Swiss study with acceptance of the price as appropriate by 87.9% of the patients [31]. The costs of 700 EUR should be set in the context of the Swiss median income of 6665 EUR/month [32].

### Strengths and limitations

Our study has several strengths. Firstly, we conducted a total of 77 follow-up interviews, thereby covering an extensive range of patients. Secondly, the mixed-methods design enabled the research team to collect quantitative and qualitative data, thereby allowing for an evaluation of the relative importance of the investigated aspects. In addition to the questions in the interview guide, patients got the opportunity to express their appraisal of the PGx service in open questions.

We acknowledge some limitations for the interpretation of our study. Firstly, the positive appraisal of the service may be influenced by a social desirability bias [33], as the PGx service as well as the follow-up interviews *F*1 and *F*2 were conducted by the same person (CJ). Secondly, we cannot exclude that patients did not remember the details of pharmacy visits 1 and 2 and the subsequent consultation with the physician, especially patients who were interviewed far more than four months after the PGx service. Initially a period of four to six months after the second visit to the pharmacy was estimated as adequate by the PGx expert team, because some patients did not arrange a consultation with the referring physician directly after the second visit and the titration of drugs (especially in psychiatry) takes time. However, with a mean time of 229 days since the 2nd visit this intent could not be met. Thirdly, we did not make any standardized measurements of health literacy, but only categorized the educational background. Fourthly, patients participating in this study were subject to a selection bias for patients receiving PGx testing and do not represent the general population. It would be valuable to also include the view of those not currently targeted for PGx testing.

### Looking forward

As cited before [13, 14], we see the pharmacist as provider of the PGx service. To collect the appraisal of referring physicians, we effected a pilot focus group with four physicians. We think that the PGx service does not only seek interprofessional collaborations but also represents a chance to enable shared decision-making. In the future, we intend to extend the interprofessional collaboration with physicians, and we also intend to assess the perspective of the pharmacist to enable a successful implementation of the PGx service.

## Conclusion

The evaluation of a new pharmacist-led service comprising PGx panel testing and counselling showed a relevant optimization of the pharmacotherapy in primary care with more than two thirds of the patients having at least one recommendation implemented. Most patients were able to understand the PGx recommendations provided by the PGx service and the usability of the documents they received was rated as very high. Overall, patient experienced a gain in medication knowledge and most of the patients were willing to pay for the PGx service.

For future PGx testing and counselling, HCPs should consider the patients’ health literacy in a standardized way and use adequate communication skills to enhance the patient's understanding in PGx and to attenuate potential negative expectations.
